# Textural Analysis of Magnetic Resonance Images as an Additional Evaluation Tool of Parotid Glands in Sjögren—Primarily Findings

**DOI:** 10.3390/biomedicines11123132

**Published:** 2023-11-24

**Authors:** Małgorzata Grzywińska, Magdalena Karwecka, Anna Pomorska, Ninela Irga-Jaworska, Dominik Świętoń

**Affiliations:** 1 Neuroinformatics and Artificial Intelligence Lab, Department of Neurophysiology, Neuropsychology and Neuroinformatics, Medical University of Gdansk, 80-210 Gdansk, Poland; 2 Department of Radiology, University Clinical Center, 80-952 Gdansk, Polanddominik.swieton@gumed.edu.pl (D.Ś.); 3 2nd Department of Pediatrics, Haemathology & Oncology, Medical University of Gdansk, 80-210 Gdansk, Poland; 4 2nd Department of Radiology, Medical University of Gdansk, 80-210 Gdansk, Poland

**Keywords:** Sjögren’s syndrome (SS), texture analysis (TA), radiomics

## Abstract

Magnetic Resonance Imaging (MRI) plays a leading role in diagnosing soft tissue pathologies, especially in the head and neck. It is increasingly popular for evaluating salivary gland issues like neoplasms and Sjogren’s Syndrome. Advanced MRI methods, including MRI sialography and texture analysis, offer non-invasive alternatives, enhancing MRI’s role. This study focused on the relationship between the apparent diffusion coefficient (ADC) and T2-weighted MRI sialography and texture analysis (TA) of parotid glands in children with and without Sjogren’s Syndrome (SS). Using 3.0 Tesla MRI with DWI and T2-weighted imaging, expended texture analysis, first-order statistics (FSOs), second-order, and higher-order statistics were conducted. The results showed significant differences in parotid ADC values, with lower values in the SS group, particularly in cases of higher disease activity. Lower kurtosis values were associated with more severe Tonami Scale grades. FSO parameters correlated well with the texture analysis from T2-weighted images, indicating promise in grading parotid gland inflammation. However, further research is needed to understand the impact of variables like binning and region of interest (ROI) size. This study highlights the potential of texture analysis for assessing parotid gland inflammation and emphasizes the need for more investigations in this area.

## 1. Introduction

The diagnosis and treatment of Sjögren’s Syndrome (SS), a complicated autoimmune illness, establish unique difficulties, especially in the case of children. Since the symptoms of SS in children are nonspecific and are usually accompanied by a delayed diagnosis, it is essential to identify the disease early and evaluate its activity. As reported in the literature [1], SS can present itself in various ways, impacting not just the salivary and lacrimal glands but also influencing other organs such as the joints, lungs, kidneys, veins, and muscles. A formal diagnosis of SS in children is complicated by the lack of SS criteria dedicated to children. The latest 2016 ACR/EULAR criteria for SS used in adults are often not adequate for the onset of disease in children [2]. Although these criteria can be used in diagnosis, the opinion of an experienced specialist dealing with patients with SS remains crucial in establishing a diagnosis of SS in children.

Considering the complexity of the illness, finding suitable and non-invasive diagnostic methods is needed. However, limited sensitivity is a common problem with conventional approaches used to diagnose SS in children, particularly those that rely on immunologic characteristics. Additionally, clinical signs may not last long.

One promising approach is imaging methods, which offer the advantage of being non-invasive and can provide additional information about the extent and characteristics of SS-related changes within the affected organs. Among these imaging techniques, salivary gland ultrasound stands out as a promising screening tool with the potential to replace invasive salivary gland biopsy in the diagnostic process [3]. However, it is important to note that the absence of a validated assessment system and the natural variability between sonographers present significant challenges in achieving objective and consistent monitoring using this method.

Another common imaging approach in SS diagnosis is sialography, which relies on radiographic strategies to observe anatomical changes in the salivary gland duct system. In contrast, scintigraphy offers valuable data by measuring the rate and intensity of technetium-99m (99mTc) uptake in the mouth after intravenous infusion, providing insights into salivary gland function. Despite their utility, both methods are invasive and carry the risk of radiation exposure side effects, making them less than ideal for pediatric patients [4].

MRI with a sialography sequence is an effective alternative method that, unfortunately, is underappreciated [5]. This approach takes advantage of the multi-parametric nature of MRI, allowing for the assessment of gland inflammation, structure, and function [6,7]. The relatively new field of the textural analysis of MR images holds promise in enhancing the diagnostic process.

Texture analysis in the context of MRI involves the quantitative assessment of spatial patterns of signal intensity changes within a specific region of interest. It comprises various techniques for quantifying the image’s gray-level patterns and voxel relationships. These textural parameters are loaded sources of information, offering insights into the region’s heterogeneity, often showing microstructural changes. Recent research suggests that individual texture characteristics can be optimally combined with dynamic disease progression indicators, offering new dimensions for diagnosing and monitoring diseases. Prior studies have demonstrated the high accuracy of texture analysis in differentiating between SS patient grades based on apparent diffusion coefficient (ADC) values in adults [8,9]; however, there remains a gap in standardized methods for texture assessment. Some studies have explored diverse approaches to analyzing MR images for diagnostic purposes in SS [10,11], emphasizing the need for a unified framework for texture assessment in the context of this challenging disease.

Sjögren’s Syndrome in pediatric patients causes unique diagnostic challenges due to its nonspecific and often delayed clinical presentation. Traditional diagnostic methods lack the sensitivity for early detection. Imaging methods, including salivary gland ultrasound, sialography, and scintigraphy, offer valuable insight but have essential limitations and risks. MRI with sialography, while effective, is underused. A texture analysis of MR images is a promising possibility for improving the diagnostic process, offering quantitative insights into SS’s microstructural and dynamic aspects.

## 2. Materials and Methods

### 2.1. Research Group

The Independent Bioethics Committee for Scientific Research at the Medical University of Gdańsk provided ethical approval for this study (NKBBN/228/2021). All participants (above 16 years) or legal guardians (under 16 years) gave written consent to participate in the examination, depending on the participant’s age, as below:

1. For participants above 16 years, informed consent was obtained from all for study participation.
2. For participants below 16 years, informed consent was obtained from all of the parents or legal guardians for study participation.

The research was conducted according to the Declaration of Helsinki of 1975, which was subsequently revised in 2000. All analysis images were obtained with consent from all of the subjects and were safeguarded throughout the study.

A total of 36 patients were included in the primary analysis, all of whom met the inclusion criteria of having a positive small labial salivary gland biopsy for Sjögren’s Syndrome (SS). These patients underwent routine clinical control. The age of the patients ranged from 5 to 20 years old. The mean age of the patient group was 12.5 years old, with a median of 12 years old. The patient group consisted of 16 males and 20 females, and the interquartile range (IQR) of their ages was 5.

Conversely, the control group included 20 children or young adolescents, with a mean age of 13.5 and a median age of 14. There were 12 males and 8 females in the control group, and the interquartile range (IQR) of their ages was 4.

The patients’ demographic information is shown in Table 1, which provides further details on the characteristics of the study participants.

### 2.2. MRI Examination

The examinations in this study were performed using a Philips Achieva 3T TX magnetic resonance scanner (Philips Healthcare, Best, The Netherlands). To enhance the imaging precision of the head–neck region assessment, a 16-channel neurovascular coil was used.

The examination protocol included a survey sequence and a set of three-plane, low-resolution, large field-of-view (FOV) images designed to precisely localize the specific anatomical region under examination. This is a crucial step in imaging to focus on the intended area of interest.

Following the survey sequence, morphological sequences were performed in three planes. This sequence provides the necessary anatomical orientation for salivary glands. It is important to highlight that these sequences were chosen for their suitability in our analysis and for clinical assessment.

The primary sequences that formed the foundation of our analysis included two essential imaging techniques:

1. Diffusion-weighted imaging (DWI) is sensitive to the movement of water molecules within tissues. It offers a valuable understanding of the tissue microstructure and helps identify areas of restricted diffusion, which can specify various pathological conditions.
2. T2-weighted imaging offers exceptional contrast for visualizing different types of tissues, making this type of sequence helpful in identifying anatomical structures and pathological changes. This image can highlight areas of edema and inflammation, which can be highly important in the context of diseases like Sjögren’s Syndrome.

The combination of these sequences (along with the other morphological imaging sequences mentioned in Table 2) formed the basis of our analysis. This allowed a comprehensive evaluation of the anatomical and pathological aspects related to the patients in the study and played a fundamental role in understanding Sjögren’s Syndrome in the pediatric population of our study.

### 2.3. Image Analysis

The MR images were reviewed by a qualified radiologist who evaluated the image findings.

The MR images were determined according to the high T2 signal intensity size through all areas of glands in the MR sialography sequence. This made it possible to evaluate the state of the salivary glands in a way that was both thorough and objective, based only on the radiological features shown in the MR images.

The Tonami modified criteria were as below [12]:

1. Grade 0 (normal)—no evidence;
2. Grade 1 (punctate)—areas ≤ 1 mm in diameter;
3. Grade 2 (globular)—1–2 mm in diameter;
4. Grade 3 (cavitary)—up to 1 cm in diameter;
5. Grade 4 (destructive)— complete destruction of the gland parenchyma.

The ADC maps (apparent diffusion coefficient maps) were automatically generated from DWI (b = 0.500 and 1000 s/mm^2^) scans by the manufacturing software (R5.3, Philips Healthcare, Best, The Netherlands), which was integrated with the workstation using the monoexponential model [11]:

$$S=S_{0}\cdot e^{-b\cdot ADC}$$

To correct the field inhomogeneity, we used a specialized correction algorithm known as the N4 algorithm [13]. This algorithm is designed to alleviate distortion caused by inhomogeneity in magnetic field strength within the image data. This can enhance the quality and accuracy of the analysis. Following the field inhomogeneity correction, a texture analysis using package pyRadiomics (v3.0.1) was performed (with specific reference to the Radiomics modules [14]). We set particular parameters, like absolute bound and bin width, to ensure the consistency and reliability of the analysis.

The region of interest (ROI) was drawn manually, covering all glands. Additionally, to minimize any potential influence from surrounding tissues or artifacts at the periphery of the glands, we kept approximately 1 mm distance from the edge when drawing the ROI. The ROI was drawn in all volumes of each parotid in ADC maps and T2 sequences.

**Figure 1:**
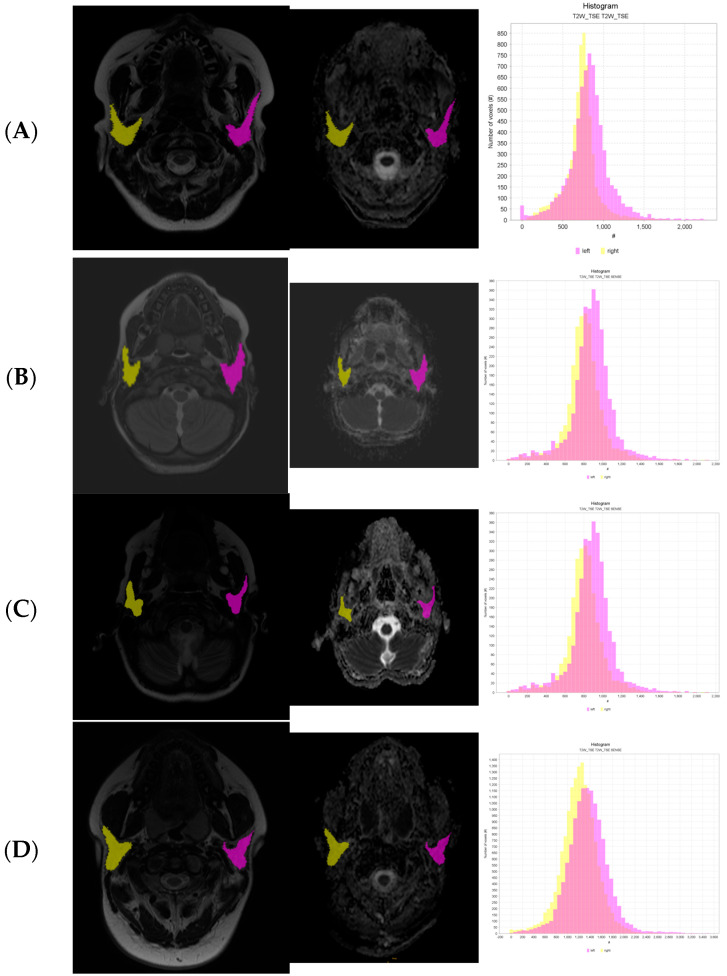
Axial images of T2-weighted imaging, diffusion-weighted imaging (DWI), and apparent diffusion coefficient (ADC). Histogram correlated with T2 images. For Tonami scale: (**A**) grade 0, (**B**) grade 1, (**C**) grade 2, (**D**) grade 3.

The texture features obtained from these regions of interest (ROIs) following formulas were divided into subgroups [15] (Figure 1):

1. First-order statistic (FSO), including Energy, Total Energy, Entropy, Minimum, 10th percentile, 90th percentile, Maximum, Mean, Median, Interquartile Range, Range, Mean Absolute Deviation (MAD), Robust Mean Absolute Deviation (rMAD), Root Mean Square (RMS), Standard Deviation, Skewness, Kurtosis, Variance, Uniformity;
2. Gray Level Co-occurrence Matrix (GLCM) features, including Joint Average, Cluster Prominence, Cluster Shade, Cluster Tendency, Contrast, Correlation, Difference Average, Difference Entropy, Difference Variance, Joint Energy, Joint Entropy, Informational Measure of Correlation 1 and 2 (IMC), Inverse Difference Moment (IDM), Maximal Correlation Coefficient (MCC), Inverse Difference Moment Normalized (IDMN), Inverse Difference (ID), Inverse Difference Normalized (IDN), Inverse Variance, Maximum Probability, Sum Average, Sum Entropy, Sum of Squares;
3. Gray Level Size Zone Matrix (GLSZM) features, including Small Area Emphasis (SAE), Large Area Emphasis (LAE), Gray Level Non-Uniformity (GLN), Gray Level Non-Uniformity Normalized (GLNN), Size-Zone Non-Uniformity (SZN), Size-Zone Non-Uniformity Normalized (SZNN), Zone Percentage (ZP), Gray Level Variance (GLV), Zone Variance (ZV), Zone Entropy (ZE), Low Gray Level Zone Emphasis (LGLZE), High Gray Level Zone Emphasis (HGLZE), Small Area Low Gray Level Emphasis (SALGLE), Small Area High Gray Level Emphasis (SAHGLE), Large Area Low Gray Level Emphasis (LALGLE), Large Area High Gray Level Emphasis (LAHGLE);
4. Gray Level Run Length Matrix (GLRLM) features, including Short Run Emphasis (SRE), Long Run Emphasis (LRE), Gray Level Non-Uniformity (GLN), Gray Level Non-Uniformity Normalized (GLNN), Run Length Non-Uniformity (RLN), Run Length Non-Uniformity Normalized (RLNN), Run Percentage (RP), Gray Level Variance (GLV), Run Variance (RV), Run Entropy (RE), Low Gray Level Run Emphasis (LGLRE), High Gray Level Run Emphasis (HGLRE), Short Run Low Gray Level Emphasis (SRLGLE), Short Run High Gray Level Emphasis (SRHGLE), Long Run Low Gray Level Emphasis (LRLGLE), Long Run High Gray Level Emphasis (LRHGLE);
5. Neighboring Gray Tone Difference Matrix (NGTDM) features, including Coarseness, Contrast, Busyness, Complexity, Strength;
6. Gray Level Dependence Matrix (GLDM) features, including Small Dependence Emphasis (SDE), Large Dependence Emphasis (LDE), Gray Level Non-Uniformity (GLN), Dependence Non-Uniformity (DN), Dependence Non-Uniformity Normalized (DNN), Gray Level Variance (GLV), Dependence Variance (DV), Dependence Entropy (DE), Low Gray Level Emphasis (LGLE), High Gray Level Emphasis (HGLE), Small Dependence Low Gray Level Emphasis (SDLGLE), Small Dependence High Gray Level Emphasis (SDHGLE), Large Dependence Low Gray Level Emphasis (LDLGLE), Large Dependence High Gray Level Emphasis (LDHGLE).

### 2.4. Statistical Analysis

A statistical analysis was performed using SPSS Statistics 27 (IBM, Armonk, NY, USA). We assessed the normality of all results, and the Shapiro–Wilk test showed that none of the parameters followed a normal distribution. Therefore, we employed the Spearman correlation test to examine the relationships between the collected data and identify correlations between texture features in diseased and healthy glands. We applied the independent-samples Kruskal–Wallis test to compare group differences among the glands. Afterward, a post hoc analysis was performed to determine which groups showed significant differences, with a significance of *p* < 0.05.

## 3. Results

### 3.1. MRI Sialography Grades by the Tonami Scale

We assessed the MRI sialography grades for each gland, considering the presence of cystic changes [12]. Our findings indicated that grade 0 was observed in 12 glands (16%), grade 1 in 8 glands (11%), grade 2 in 30 glands (42%), and grade 3 in 22 glands (31%) (Table 1).

### 3.2. ADC Values

**Figure 2:**
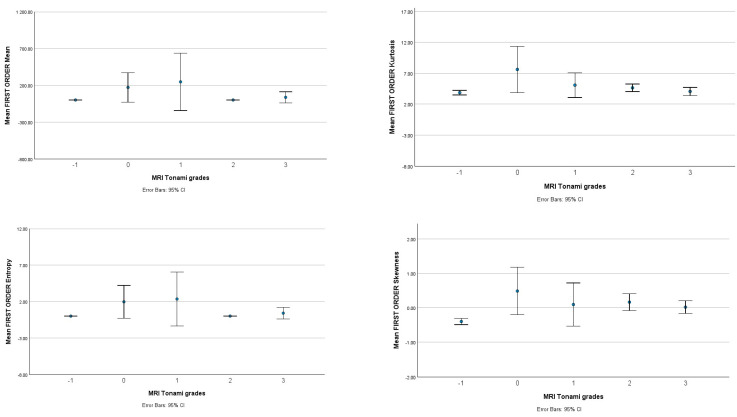
Graphic presents the value of first-order statistics from ADC depending on MRI Tonami’s grades.

The value of the ADC from Sjögren’s Syndrome patients and the control group was obtained from the ROI in each gland; the mean ADC value depending on the MRI morphology grade is present in Table 3 (grade 0—0.88 ± 0.14; grade 1—0.86 ± 0.09; grade 2—0.97 ± 0.17; grade 3—1.04 ± 0.19 [10^−3^ mm$\frac{2}{s}$]). The ADC value from the control group: 1.04 ± 0.10 [10^−3^ mm$\frac{2}{s}$]. There was a significant difference (using the independent-samples Kruskal–Wallis test: ꭓ^2^(4) = 25.139, *p* < 0.001) of the ADC value relative to MRI morphology grades (Tonami scale [12]) (Figure 2). Therefore, further analyses were performed with a pairwise comparison with Bonferroni correction. An obtained significant difference (*p* < 0.05) between groups (Table 3) was

- 1–healthy (*p* = 0.005),
- 1–3 (*p* = 0.046),
- 0–3 (*p* = 0.035),
- 0–healthy (*p* = 0.001).

**Table 3:** Mean values of image and texture analysis of ADC images compared to MRI Tonami grades.

Parameters	Tonami Scale									
	Healthy		0		1		2		3	
	Mean	Std. Deviation	Mean	Std. Deviation	Mean	Std. Deviation	Mean	Std. Deviation	Mean	Std. Deviation
Mean [10^−3^ mm^2^/s]	1.04	0.10	0.88	0.14	0.86	0.09	0.97	0.17	1.04	0.19
Median [10^−3^ mm^2^/s]	1.06	0.10	0.86	0.14	0.88	0.09	0.96	0.17	1.03	0.19
Entropy	0.01	0.00	2.95	4.05	5.46	4.74	0.04	0.01	0.97	0.27
Kurtosis	3.86	1.19	9.24	6.63	6.48	1.97	5.03	0.35	1.23	0.17
Skewness	−0.39	0.28	0.99	0.09	0.57	0.07	0.36	0.07	0.17	0.03

### 3.3. Whole-Volume ADC Image for Texture Analysis

A whole-volume ADC analysis was computed for each gland without distinguishing between the left and right glands. As presented in Table 3, a moderate correlation was observed between texture features and MRI morphology grades. Notably, a prevalent positive correlation was found between MRI-positive sialography and nearly every texture parameter with higher statistical attributes. On the contrary, a negative correlation was observed for first-order statistics (FSOs), including skewness, kurtosis, and entropy.

The parotid ADC values from the whole-volume region of interest (ROI) were significantly lower in group 1 on the Tonami scale than in groups with more advanced changes. This difference was confirmed by a Kruskal–Wallis H test, demonstrating statistical significance (*p* > 0.05). Furthermore, pairwise comparisons of the Tonami Scale for groups 1–2 and 1–3 revealed significant differences (as indicated in Table 3). Notably, the most critical finding in this research is the statistically significant difference between grade 0 and the healthy group and grade 3, with significance noted as *p* < 0.05.

### 3.4. Whole-Volume T2 for Texture Analysis

Each salivary gland was analyzed without differentiating between the left and right glands. This method made it possible to thoroughly evaluate the texture properties and how they relate to the MRI morphological grades. Table 4 summarizes the findings and indicates a moderate correlation between the MRI morphological grades and the obtained texture features. This result suggests that the textural characteristics obtained from the imaging data are meaningful and can highlight the structural and compositional changes inside the salivary glands.

A positive and moderate correlation was found between individual MRI morphology parameters and some specific texture parameters, specifically the first-order statistics (FSOs) parameters (Table 4). This positive correlation indicates that the FSO parameters, which characterize basic statistical features of the images, can effectively capture significant details about the structural features of the gland, as seen in the MR images.

## 4. Discussion

The texture analysis of images is a complex process that involves the assessment of various spatial relationships and characteristics within the image. The set of texture features we have examined, including Small Dependence Emphasis (SDE), Large Dependence Emphasis (LDE), Gray Level Non-Uniformity (GLN), Dependence Non-Uniformity (DN), Dependence Non-Uniformity Normalized (DNN), Gray Level Variance (GLV), Dependence Variance (DV), Dependence Entropy (DE), Low Gray Level Emphasis (LGLE), High Gray Level Emphasis (HGLE), Small Dependence Low Gray Level Emphasis (SDLGLE), Small Dependence High Gray Level Emphasis (SDHGLE), Large Dependence Low Gray Level Emphasis (LDLGLE), and Large Dependence High Gray Level Emphasis (LDHGLE), collectively provide valuable insights into the textural characteristics of an image.

This primary study examines salivary gland texture in MR images within a pediatric Sjogren Syndrome (SS) cohort. The research investigates the differences in textural characteristics within two regularly acquired MR images in SS patients—ADC maps and T2-weighted images. In total, 36 patients were included in the analysis, with each salivary gland analyzed individually (N = 72). For the control group, 20 examinations were conducted, examining 40 glands.

A critical objective of this study was to identify the most suitable MR image type for grading disease severity using texture analysis. The Tonami scale for MRI positive sialography served as the standard for comparing data derived from texture analysis. Information was extracted from the intensity of each voxel within the region of interest (ROI) covering the entire gland volume (with the 1 mm margin).

The parotid ADC values from whole volume ROIs were significantly lower in the Tonami scale grades 0 and 1 than in the high-activity group (grade 3 and above). Notably, the average ADC value in the reference group displayed a significant difference to grade 1. This could suggest that the ADC, often used as an inflammatory marker, can help in promptly diagnosing early-stage SS (grade 1). These findings indicate that higher sialography grades correspond to elevated ADC values [9,16,17].

Moreover, lower kurtosis and skewness values were observed in the highest Tonami Scale grade, which is recognized to likely increase tissue heterogeneity due to atrophy, fibrotic tissue replacement, and fluid-filled sialectasis. This condition influences the tissue structure, resulting in advanced changes. Unlike previous studies [10,18,19] in adults, unchanged or minimally affected glands in MRI sialography exhibited a notable decrease in ADC values, which is indicative of an ongoing inflammatory process preceding structural remodeling (Figure 2 and Table 5). This discovery reinforces the ability of an early-stage diagnosis of pediatric SS, which involves a particularly challenging population [2].

In the T2 image texture analysis, the first-order statistic (FSO) parameters displayed a moderate correlation among individual parameters. However, lower kurtosis values were observed in the highest activity SS group on the Tonami Scale. Entropy values remained relatively consistent across all grades, potentially due to lower sensitivity to inflammation than the DWI sequence. Nevertheless, this characteristic upholds high repeatability and minimizes artifacts, benefiting this sequence more. A T2-weighted MR image signal intensity primarily decreases from intracellular and extravascular space, showing acute inflammation which is often associated with SS disease activity [10,18].

These texture features collectively offer a comprehensive tool for quantifying and understanding the textural content of images, allowing their application in medical imaging for tasks ranging from quality control to disease diagnosis. The choice of which features to use will depend on the specific image analysis task and the nature of the texture patterns of interest [20,21,22,23].

Despite these insightful findings, several limitations need consideration. The small sample size in this, inherent to the pediatric nature of the group, should be acknowledged. Ensuring an optimal MRI protocol and addressing field inhomogeneity correction are crucial. Additionally, future studies should explore excluding signals from cysts in high-grade SS, which can artificially elevate ADC map signals. These limitations need further investigation.

Moreover, the potential link between histological activity and basic, unfiltered texture parameters on T2-weighted images and ADC maps may be fundamental to evaluating disease activity. Combining both sequences could address issues concerning signal-to-noise ratio and volume averaging in ADC maps. Textural analysis alongside sialography sequences and ADC maps presents a novel approach to early-stage SS diagnosis in children and improves disease monitoring.

The recent review of Wenxing Zhong et al. [24] emphasizes the efficacy of magnetic resonance imaging (MRI) in evaluating gland structure, size, and abnormalities such as atrophy, inflammation, and fibrosis. MRI plays a role in disease monitoring and treatment assessment, particularly with recent techniques like dynamic contrast-enhanced MRI, diffusion-weighted imaging, and radiomics. They reference the adult study by Muntean et al., which reported a 91% sensitivity and 83% specificity in radiomics analysis [25]. In our opinion, MRI might find a place in the diagnostic criteria of SS, especially in the pediatric population. According to our results, MRI improves diagnostic precision in diagnosing early stages of the disease, potentially eliminating biopsy from the diagnostic algorithm. Before inclusion, it would be necessary to carefully evaluate standardization, repeatability, and association with clinical results in larger groups.

It is worth mentioning the historical context of Sjögren’s Syndrome and Mikulicz Syndrome; the conventional approach to managing these conditions involved bilateral parotidectomy as a primary solution. However, this method occasionally led to complications such as Frey syndrome, characterized by gustatory sweating. Notably, Freni et al. [26] demonstrated the successful use of botulinum toxin in addressing Frey syndrome, offering an alternative to the traditional surgical approach [26,27]. Finding a way to improve disease monitoring and prevent invasive treatment methods is crucial. Nevertheless, in the case of children who do not exhibit dryness symptoms, objective dryness, or SS antibodies, an additional investigation, including salivary gland imaging and histopathological assessment, must assist in confirming the diagnosis [4]. It suggests that many children with recurrent or persistent salivary gland enlargement of unknown origin are likely to receive an SS diagnosis following a comprehensive workup. However, not meeting existing adult SS criteria does not exclude the diagnosis of SS [2,28]. Continued observation with periodic repeat testing (imaging, serological, functional) to evaluate for progression to SS is crucial for these children.

Further research and cooperative efforts are reasonable to explore the feasibility and reliability of integrating MRI findings into the diagnostic framework for SS [25]. The future of textural analyses of salivary glands in Sjögren’s Syndrome could be linked to artificial intelligence (AI). It could hold significant promise for advancing research and clinical applications, contributing first to segmentation and performing detailed quantitative analyses of textural features in salivary gland images. AI can provide a more objective and consistent analysis, contributing to a deeper understanding of Sjögren’s Syndrome manifestations. The other side of using AI is pattern recognition, but it will require a large dataset to recognize these patterns. Furthermore, AI has the potential to be one of the most important aspects in accelerating the pace of research and enhancing diagnostic accuracy.

## 5. Conclusions

The complex nature of Sjögren’s Syndrome, its various clinical presentations, and the difficulties related to traditional diagnostic methods highlight the significance of investigating innovative approaches like textural analyses of MRI.

This study shows different areas in which textural analysis can improved, like:

1. Early diagnosis of Sjögren’s Syndrome (grade 1): One of the most significant implications of this study is the prospect of early diagnosis of Sjögren’s Syndrome, especially in children. This is crucial because Sjögren’s Syndrome is known for its nonspecific symptoms in the early stage of inflammation. Using MRI-based texture analysis, we can detect subtle changes at a microstructural level within parotid glands, which allows us to identify Sjögren’s Syndrome in the early stages. This can lead to prompt intervention and a better prognosis for young patients.
2. Monitoring of remodeling salivary glands in the process of Sjögren’s Syndrome: Because Sjögren’s Syndrome is characterized by the progressive remodeling and destruction of the salivary glands, texture analysis can be a valuable tool for monitoring these changes over time by quantifying the heterogeneity and eventual microstructural changes in the glands. Our study suggests that we can track the disease’s progression and possible impact on the glandular tissue. This information will significantly impact the individual treatment strategy and therapeutic intervention.
3. Reducing radiation exposure: Traditional diagnostics like sialography and scintigraphy can be replaced by magnetic resonance imaging only. This can be a safer approach for the pediatric population.
4. Treatment planning can be more individualized: The ability to assess microstructures through textural analysis can be essential for better understanding the characteristics of each patient’s condition.

In conclusion, textural analyses of MR images for evaluating Sjögren’s Syndrome hold great promise, especially in childrens’ diagnostics. It has the potential to offer a non-invasive, precise, and patient-centric approach. This primary research explores the full potential of textural analysis and defines our understanding of its significance in clinical practice.

## Figures and Tables

**Table 1:** The demographic characteristics of patients.

Index		Value SS Group	Value Healthy Group
Age	n.a.*	12.5 ± 3.4 yrs	13.5 ± 2.4 yrs
Gender	female	20	8
	male	16	12
Tonami scale	0	12	n.a.
	1	8	n.a.
	2	30	n.a.
	3	22	n.a.

* n.a.—not applicable.

**Table 2:** MRI protocol.

Sequence	FOV (mm)	Slice Thickness/Gap (mm)	Voxel (mm)	Suppress Fat	Flip Angle	Number of Averages	TE/TR (ms)	Matrix	Plane
Survey/localizer	250/250	10	0.98 × 1.95 × 10	-	15	1	4.6/11	256	Coronal, transverse, sagittal
T2_TSE	210/210	3/0.5	0.80 × 1.15 × 3	-	90	2	90/2500	512	Coronal
T2_TSE	240/240	3/0.6	0.70 × 0.87 × 3	-	90	2	90/3000–4500	640	Transverse
T2_STIR	210/210	3/0.6	0.78 × 0.98 × 3	STIR	-	2	90/1400–4000	560	Transverse
3D_TSE_SPIR_obligue	300/245/48	2	1 × 1.24 × 2	SPIR	90	2	90/120	1008	Sagittal
mDIXON	250/250/130	1	1 × 1 × 2	-	10	3	Shortest/shortest	256	Transverse
DWI	240/240	4.5/1	1.5 × 2.18 × 4.5	SPIR	90	1	Shortest/shortest	288	Transverse

TSE—Turbo Spin Echo; SPAIR—Spectral Presaturation with Inversion Recovery; 3D TSE SPIR imaging—3D Turbo Spin Echo Spectral Presaturation with Inversion Recovery imaging—Sialography; mDixon—time-consuming acquisition of in-phase and opposed-phase gradient-echo images; STIR—Short-TI Inversion Recovery; T2—the time constant for decay/dephasing of transverse magnetization; DWI—Diffusion-weighted Imaging.

**Table 4:** Mean values of image and texture analysis of T2 images compared to MRI Tonami grades.

Texture Parameters	Tonami Scale									
	Healthy		0		1		2		3	
	Mean	Std. Deviation	Mean	Std. Deviation	Mean	Std. Deviation	Mean	Std. Deviation	Mean	Std. Deviation
Median	891.53	244.23	698.58	252.43	890.44	481.32	849.78	344.92	897.32	300.99
Entropy	7.51	1.76	8.21	0.44	8.48	0.49	8.49	0.69	8.72	0.70
Kurtosis	4.79	1.57	7.33	3.58	4.89	0.68	6.02	1.56	5.24	1.78
Mean	887.56	242.88	697.16	246.50	880.58	451.04	850.80	345.87	911.03	303.55
GLDM_Dependence NonUniformity	5791	3201	13,600	669	11,600	2390	13,400	7720	14,200	6530
GLDM_Small Dependence High Gray Level Emphasis	95,569	563,232	115,000	887,000	203,000	202,000	191,000	162,000	209,000	144,000
GLCM_Imc1	−0.32	0.09	−0.27	0.06	−0.31	0.07	−0.32	0.08	−0.34	0.06
GLRLM_Run Entropy	7.67	1.21	8.26	0.43	8.53	0.48	8.53	0.67	8.76	0.68
GLSZM_Zone Entropy	8.11	1.19	8.69	0.38	8.89	0.37	8.92	0.54	9.07	0.58

**Table 5:** Pairwise comparisons of ADC values with MRI Tonami grades.

Sample 1–Sample 2	Test Statistic	Std. Error	Std. Test Statistic	Sig.	Adj. Sig. ^a^
1–3	−39.756	14.033	−2.833	0.005	0.046
1–healthy	46.178	13.180	3.504	<0.001	0.005
0–3	−33.905	11.605	−2.922	0.003	0.035
0–healthy	40.327	10.558	3.820	<0.001	0.001

Each row tests the null hypothesis that the Sample 1 and Sample 2 distributions are the same. Asymptotic significances (2-sided tests) are displayed. The significance level is 0.050. ^a^ Significance values have been adjusted by the Bonferroni correction for multiple tests.

## Data Availability

The data presented in this study are available on reasonable and qualified research request from the corresponding author. Data requestors will need to sign a data access agreement.
